# Detecting Mechanisms of Karyotype Evolution in *Heterotaxis* (Orchidaceae)

**DOI:** 10.1371/journal.pone.0165960

**Published:** 2016-11-10

**Authors:** Ana Paula Moraes, André Olmos Simões, Dario Isidro Ojeda Alayon, Fábio de Barros, Eliana Regina Forni-Martins

**Affiliations:** 1 Departamento de Biologia Vegetal, Instituto de Biologia, Universidade Estadual de Campinas/UNICAMP, Campinas, São Paulo, Brasil; 2 Departamento de Genética, Instituto de Biociências, Universidade Estadual Paulista/UNESP, Rubião Júnior, Botucatu, São Paulo, Brasil; 3 Instituto de Ciência e Tecnologia, Universidade Federal de São Paulo/UNIFESP, São José dos Campos, São Paulo, Brasil; 4 The Biodiversity Research Center and Department of Botany, University of British Columbia, Vancouver, Canada; 5 Instituto de Botânica, São Paulo, Brasil; Università di Pisa, ITALY

## Abstract

The karyotype is shaped by different chromosome rearrangements during species evolution. However, determining which rearrangements are responsible for karyotype changes is a challenging task and the combination of a robust phylogeny with refined karyotype characterization, GS measurements and bioinformatic modelling is necessary. Here, this approach was applied in *Heterotaxis* to determine what chromosome rearrangements were responsible for the dysploidy variation. We used two datasets (nrDNA and cpDNA, both under MP and BI) to infer the phylogenetic relationships among *Heterotaxis* species and the closely related genera *Nitidobulbon* and *Ornithidium*. Such phylogenies were used as framework to infer how karyotype evolution occurred using statistical methods. The nrDNA recovered *Ornithidium*, *Nitidobulbon* and *Heterotaxis* as monophyletic under both MP and BI; while cpDNA could not completely separate the three genera under both methods. Based on the GS, we recovered two groups within *Heterotaxis*: (1) "small GS", corresponding to the Sessilis grade, composed of plants with smaller genomes and smaller morphological structure, and (2) "large GS", corresponding to the Discolor clade, composed of plants with large genomes and robust morphological structures. The robust karyotype modeling, using both nrDNA phylogenies, allowed us to infer that the ancestral *Heterotaxis* karyotype presented 2*n* = 40, probably with a proximal 45S rDNA on a metacentric chromosome pair. The chromosome number variation was caused by ascending dysploidy (chromosome fission involving the proximal 45S rDNA site resulting in two acrocentric chromosome pairs holding a terminal 45S rDNA), with subsequent descending dysploidy (fusion) in two species, *H*. *maleolens* and *H*. *sessilis*. However, besides dysploidy, our analysis detected another important chromosome rearrangement in the Orchidaceae: chromosome inversion, that promoted 5S rDNA site duplication and relocation.

## Introduction

The karyotype, *i*.*e*., the complete eukaryotic chromosome complement, was shaped during species evolution through chromosome rearrangements [1–8]. Fusion and fission are two of the most important chromosome rearrangements causing dysploidy; *i*.*e*., the variation in chromosome number due to rearrangements without any gain or loss of genetic material [9–11]. Some hypotheses have been proposed regarding the importance of fusion and fission in karyotype evolution: whereas some authors claim fusion as the most important type of rearrangement, since truly telocentric chromosomes either do not exist or are very rare [12]; others have suggested centric fission as the main process, as it minimizes the genetic risks due to deleterious reciprocal translocations, as postulated by the Minimal Interaction Theory [13–15]. So far, these hypotheses have rarely been tested in a phylogenetic framework (but see [11, 16]).

Elucidating karyotype evolutionary history is often challenging because the successive accumulation of chromosome rearrangements can obscure the order of events that have occurred across a lineage [17]. However, the use of chromosome number and other karyotype traits, such as chromosome morphology and the localization of heterochromatic bands and rDNA sites, within a phylogenetic framework, can help to reveal karyotype modifications that occurred during species evolution.

**Fig 1:**
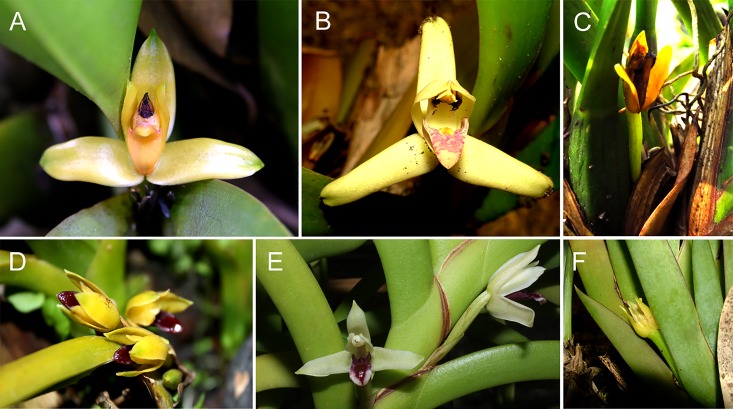
*Heterotaxis* flowers. **A**, *H*. *brasiliensis*; **B**, *H*. *violaceopunctata;* **C**, *H*. *villosa;* **D**, *H*. *superflua;* **E**, *H*. *equitans*; **F**, *H*. *valenzuelana*. Photos by A. P. Moraes.

Orchids are good models for testing the fusion/fission hypotheses due to the frequent dysploidy variation documented in different genera. Among them, *Heterotaxis* Lindl., which comprises 13 species (Fig 1) [18], presents dysploid variation, between 2*n* = 40 and 42 among the six species with known chromosome numbers (Table 1). However, only one species–*H*. *discolor* [cited as *Maxillaria discolor* (Lodd. ex Lindl.) Rchb. f.]–has been analysed for additional chromosome markers, such as heterochromatic bands and rDNA sites [19].

**Table 1:** *Heterotaxis* chromosome numbers.

Species	2*n*	*n*	Reference
*Heterotaxis discolor* (Lodd. ex Lindl.) Ojeda & Carnevali	42		[18][19]
*H*. *maleolens* (Schltr.) Ojeda & Carnevali	40		[20]
*H*. *valenzuelana* (A. Rich.) Ojeda & Carnevali	40		[20]
*H*. *villosa* Barb. Rodr.		20	[21]
*H*. *sessilis* (Lindl.) F. Barros		20	[21]
*H*. *violaceopunctata* (Rchb. f.) F. Barros	42		[18]

Morphologically, *Heterotaxis* is composed mainly of sympodial species with short rhizomes and laterally compressed, oblong unifoliate pseudobulbs, subtended by various leaf-bearing sheaths [18]. The only exceptions are *H*. *equitans* [= *Maxillaria equitans* (Schltr.) Garay] (Fig 1E) and *H*. *valenzuelana* [= *Maxillaria valenzuelana* (A. Rich.) Nash] (Fig 1F), which have a pseudomonopodial growth habit without pseudobulbs [22]. These two species were originally placed in *Marsupiaria* Hoehne, but they are currently classified as *Heterotaxis*, along with four other Brazilian *Maxillaria* Ruiz & Pav. species transferred to *Heterotaxis*: *H*. *sessilis*, *H*. *superflua*, *H*. *villosa* and *H*. *violaceopunctata* [23].

Traditionally, cytogenetic data have been superimposed onto phylogenetic trees to identify chromosome rearrangements throughout the evolution of the karyotype [24–25]. However, statistical approaches, as ancestral state reconstruction based on maximum likelihood, have recently been applied to infer karyotype evolution in a phylogenetic framework [11, 16, 26–28]. In such approaches, the phylogenetical proposals should present all species with karyotype data available and, when nuclear and chloroplast phylogenetic proposals are conflicting, both should be tested independently to reflect the more robust answer about karyotype evolution.

In order to determine which chromosome rearrangement is responsible by the dysploidy variation detected in *Heterotaxis* we aim:

(1^st^) amplify the knowledge about karyotype differences among *Heterotaxis* species based on chromosome number, heterochromatic blocks (number, distribution and type, *i*.*e*., CG-rich or AT-rich), rDNA (number and distribution of loci) and genome size (GS);

(2^nd^) build a phylogenetic framework based on *Heterotaxis* and close genera, *Nitidobulbon* and *Ornithidium*, using DNA sequence data (nuclear and chloroplast);

(3^rd^) implement model-based phylogenetic approaches to infer the chromosomal rearrangements responsible by chromosome number changes among *Heterotaxis* species. Aiming to get a robust answer about chromosome evolution, the two datasets, *i*.*e*., nuclear DNA and chloroplast DNA (nrDNA and cpDNA, respectively), were analysed separately and used comparatively as phylogenetic framework to model the karyotype evolution in *Heterotaxis*.

## Materials and Methods

### Taxon sampling

Efforts were made to sample the largest possible number of species for each analysis. A list of voucher information for all methodologies is provided in Table 2. In the subsection “Phylogenetic analyses”, 11 out the 13 *Heterotaxis* species, the three species of *Nitidobulbon* and four out the 35 of *Ornithidium* species were analysed. In the subsections “Chromosome analyses” and “Genome size estimation”, we used the six available Brazilian species of *Heterotaxis*, plus *Mapinguari desvauxiana* (Rchb.f.) Carnevali & R.B.Singer.

**Table 2:** Taxon sampling for all performed analyses. The total number of species in each genus is presented in parenthesis after the genus identification. Voucher and origin are supplied for each specimen analysed for molecular psequences (nrDNA and cpDNA), karyotype and genome size approach.

Genus	Species	Molecular1			Karyotype			Genome Size	
		Voucher		Origin	Voucher	Origin		Voucher	Origin
*Heterotaxis* Lindl. (13 species)									
	*H*. *brasiliensis* (Brieger & Illg) F.Barros								
		Koehler 0150		Brazil	AP 17	Ubatuba, Brazil		AP 17	Ubatuba, Brazil
					IBt 3244	Paraty, Brazil		IBt 3244	Paraty, Brazil
					FZB 774	Cultivated		IBt 322	Brazil
								IBt 13159	Brazil
								IBt 4107	Brazil
	*H*. *discolor* (Lodd. ex Lindl.) Ojeda & Carnevali								
		Koehler 0311		Brazil	-	-		-	-
	*H*. *equitans* (Schltr.) Ojeda & Carnevali								
		Koehler 0141		Brazil	-			-	
		IBt 979P		Brazil	IBt 979P	Brazil		IBt 979P	Brazil
		IBt 3931P		Belém, Brazil	IBt 3931P	Belém, Brazil		IBt 3931P	Belém, Brazil
	*H*. *fritzii* Ojeda & Carnevali								
		Whitten 2672		Colombia	-	-		-	-
	*H*. *maleolens* (Schltr.) Ojeda & Carnevali								
		Atwood & Whitten 5055		Honduras	-	-		-	-
	*H*. *santanae* (Carnevali & I. Ramírez) Ojeda & Carnevali								
		Whitten 6725		Ecuador	-	-		-	-
	*H*. *sessilis* (Lindl.) F. Barros								
		Atwood & Whitten 5065		Jamaica	-	-		-	-
	*H*. *superflua* (Rchb.f.) F. Barros								
		Koehler 0153		Brazil	IBt 2336	Juruena, Brazil		IBt 2336	Juruena, Brazil
					AP 76	Manaus, Brazil		AP 76	Manaus, Brazil
	*H*. *valenzuelana* (A.Rich.) Ojeda & Carnevali								
		Koehler 0263		Brazil	IBt 3177	Serra dos Órgãos, Brazil		IBt 3177	Serra dos Órgãos, Brazil
					IBt A457	Camanducaia, Brazil		IBt A457	Camanducaia, Brazil
					IBt A843	Cananéia, Brazil		IBt A843	Cananéia, Brazil
	*H*. *villosa* (Barb. Rodr.) F. Barros								
		Koehler 0367		Brazil	IBt 3934P	Belém, Brazil		IBt 3934P	Belém, Brazil
	*H*. *violaceopunctata* (Rchb.f.) F.Barros								
		Koehler 0129		Brazil	IBt 10110	Rondonia, Brazil		IBt 10111	Rondonia, Brazil
					IBt 11518	Cultivated		IBt 11518	Cultivated
								IBt 11519	Cultivated
								IBt 1713	Brazil
*Nitidobulbon* Ojeda, Carnevali & GARomero (3 species)									
	*N*. *cymbidiodes* (Dodson, J.T. Atwood & Carnevali) Ojeda & G.A. Romero								
		Atwood & Whitten 5067		Ecuador	-	-		-	-
	*N*. *nasutum* (Rchb. f.) Ojeda & Carnevali								
		Koehler 0261		Brazil	-	-		-	-
	*N*. *proboscideum* (Rchb. f.) Ojeda & Carnevali								
		Atwood & Whitten 5056		Venezuela	-	-		-	-
*Ornithidium* Salisb. ex. R.Br. (35 species)									
	*O*. *adendrobium* (Rchb. f.) M.A. Blanco & Ojeda								
		Dressler 4231		Panama	-	-		-	-
	*O*. *coccinea* (Jacq.) Salisb. ex R. Br.								
		Atwood & Whitten 5092		Puerto Rico	-	-		-	-
	*O*. *conduplicatum* Ames & C. Schweinf.								
		Blanco 1660		Costa Rica	-	-		-	-
	*O*. *fulgens* Rchb. f.								
		Whitten 2630		Panama	-	-		-	-
*Outgroup*									
	*Brasiliorchis picta* (Hook.) R.B. Singer, S. Koehler & Carnevali								
		Koehler 0337	Brazil		-	-		-	-
	*Cryptocentrum latifolium* Schltr.								
		Whitten 2349		Ecuador	-	-		-	-
	*Inti bicallosa* (Rchb.f.) M.A.Blanco								
		Whitten 2748		Ecuador	-	-		-	-
	*Mapinguari desvauxiana* (Rchb. f.) Carnevali & R.B. Singer								
		Koehler 1585		Brazil	-	-		IBt 2367	Paraty, Brazil
								IBt 3961	Jeriquara, Brazil
								IBt 4119	Peruíbe, Brazil
								IBt 807	Cananéia, Brazil
	*Xylobium zarumense* Dodson								
		Whitten 1881		Ecuador	-	-		-	-

1 –All data used in Phylogeny analysis were published by [29] and downloaded from GenBank (S2 Table), except the sequences for *H*. *equitans* IBt 979P and IBt3931P that were obtained here. Collection: FZB—Fundação ZooBotânica de Porto Alegre/RS, Brazil; IBt—Instituto de Botânica de São Paulo/SP, Brazil; AP–plants collected by Ana Paula Moraes with field study authorization by SISBIO/Brazil (37013–1 and 37417–1).

All specimens, but AP16 and AP46, were held in two living orchid collections available in Brazil (São Paulo Botany Institute—IBt—and Botanical Garden of Porto Alegre—FZB) (Table 2). The two specimens sampled from the field were collected in unprotected area and the authorization was emitted by SISBIO/Brazil (37013–1 and 37417–1).

### Phylogenetic analyses

Phylogenetic analyses based on ITS, *mat*K + *trn*K and *atp*B—*rbc*L spacer used sequences published by [29] and new sequences obtained for *H*. *equitans* (S1 Table). The species *Xylobium zarumense* Dodson, *Inti bicalosa* (Rchb.f.) M.A.Blanco and *Cryptocentrum latifolium* Schltr. were used as outgroup, following Ojeda *et al*. [30], plus *Brasiliorchis picta* (Hook.) R.B.Singer, S.Koehler & Carnevali and *Mapinguari desvauxiana—*both species previously considered *Maxillaria*.

The analyses were performed using the maximum parsimony (MP) criterion implemented in PAUP 4.0 [31] and Bayesian inference (BI) with MrBayes v.3.1.2 [32]. Both analyses were conducted on two separate matrices: (1) nrDNA and (2) cpDNA. All characters were considered unordered and equally weighted.

For the MP analysis, a heuristic search for the most parsimonious trees (MPT) included: (1) an initial round of tree searches with 1000 random addition sequence replicates (RASR), holding 10 trees at each step, and (2) tree bisection-reconnection (TBR) branch swapping with MULTREES, with steepest descent option in effect, saving a maximum of 50 trees at each replicate. All the shortest trees retained in memory were then included in a second round of searches involving exhaustive TBR branch swapping. Bootstrap support [33] was performed on each analysis using the program TreeRot v.2 [34]. Bootstrap values (BS) were evaluated as providing either moderate (50–74%) or strongly supported (75–100%).

For the BI analysis, the optimal model of sequence evolution for each molecular dataset was selected using jModeltest v.2.1.1 [35]. The Bayesian information criterion (BIC) implemented in jModeltest was used to choose the best-fitting evolutionary model for each sequence partition. Starting model parameters were assigned as uniform prior probabilities and further estimated during the analysis by allowing them to vary independently among data partitions. Twenty million generations were run using one cold and three incrementally heated Markov Chain Monte Carlo (MCMC) (Temp = 0.2), with parameters sampled every 2,000 generations. Two independent runs (Nruns = 2), starting from different random trees, were performed to ensure that the individual runs had converged to the same result. Log files were analysed with Tracer v.1.5 [36] to assess convergence and ensure that the MCMC had run long enough to obtain a valid estimate of the parameters. Based on inspection of the likelihood scores for each generation, the first 2,500 sampled generations were considered as burn-in and discarded from subsequent analyses. The post burn-in trees were imported into Tree Annotator v.1.5.4 [37], and a 50% majority-rule consensus tree was then reconstructed to obtain posterior probabilities of the clades. The majority-rule consensus tree was then analysed and edited into FigTree v.1.3.1. [38]. Posterior probabilities (PP) were considered strongly supported when equal to or higher than 0.95.

### Chromosome analysis

Root tips were pre-treated in 8-hydroxyquinoline (0.002 M) for 24 h at 10°C, fixed in absolute ethanol:glacial acetic acid (3:1, v/v) for 24 h at room temperature, and stored at -20°C. The meristems were washed in distilled water and digested in 2% (w/v) cellulase (Onozuka) / 20% (v/v) pectinase (Sigma) / 1% macerozyme (Sigma) solution and squashed in a drop of 45% acetic acid. The cover slip was removed in liquid nitrogen. For chromosome banding, preparations aged for three days were stained with CMA (0.5 mg ml^-1^) for 1 h and counterstained with DAPI (1 mg ml^-1^) for 30 min. The slides were examined using a Leica DMRA2 epifluorescence microscope, photographed with a Leica camera, and analysed using Leica LAS 3.6 software. The best slides were distained in alcohol and stored for FISH analysis. Images were processed uniformly for colour balance, contrast and brightness using Adobe Photoshop CS5 (Adobe Systems, Inc.).

For *in situ* hybridization, a D2 probe from *Lotus japonicus* (Regel) K. Larsen [39] and an R2 probe from *Arabidopsis thaliana* (L.) Heynh. [40] were used to localize 5S and 45S rDNA, respectively. The 5S rDNA probe was labelled with digoxigenin-11-dUTP and the 45S rDNA probe with biotin-14-dUPT. In both cases, nick translation (Roche Biochemicals) was performed. The *in situ* hybridization mixture was composed of 50% (v/v) formamide, 10% (w/v) dextran sulphate and 0.1% (w/v) sodium dodecyl sulphate in 2 × saline-sodium citrate buffer (SSC) with 3–5 ng ml^-1^ of each probe. The 5S rDNA probe was detected with anti-digoxigenin conjugated to rhodamine (Roche Biochemicals), and the 45S rDNA probe was detected using an avidin-FITC conjugate (Roche Biochemicals). All slides were counterstained with 2 μg ml^-1^ DAPI in Vectashield mounting medium (Vector Laboratories). Metaphase images were obtained and processed as described above under "Chromosome banding".

### Genome size estimation

To determine the DNA content of *Heterotaxis* species, approximately 25 mg of leaf tissue of each species was macerated with the same mass of the internal reference standard *Zea mays* L. cv. CE-777 (2C = 5.43 pg) [41]. The material was macerated in 1 ml of cold Tris buffer, using a scalpel blade to release the nuclei into suspension [42]. Nuclei were stained by adding 25 uL of a 1 mg ml^-1^ solution of propidium iodide (PI, Sigma^®^, USA). Additionally, 12.5 uL of RNase (2 μg ml^-1^) was added to each sample. The analysis was performed using the FACSCanto II cytometer (Becton Dickinson, San Jose, CA, USA), kindly made available by the Microbiology and Immunology Department of IBB-UNESP/Botucatu, Brazil. The histograms were obtained with FACSDiva software based on 20,000 events. A statistical evaluation was performed using the Flowing Software 2.5.1 (http://www.flowingsoftware.com/). One to five samples from each species were analysed twice, according to collection availability (Table 2). The GS obtained from each species were compared statistically by ANOVA followed by Tukey’s test using BioEstat v.5.3 [43].

### Ancestral state reconstruction of chromosome number

Ancestral state reconstruction for base chromosome number was performed with ChromEvol v.2.0 [44–45] to identify which chromosome rearrangement–fission or fusion–was responsible for chromosome number variation in *Heterotaxis*. We considered the basic chromosome number (*x*) as the haploid chromosome number that most parsimoniously explain the chromosomal variability in the group and shows a clear relationship with the basic number of the closest related groups [7, 46]. We are aware of Peruzzi (2013) [47] who, after an extensive revision about the concept of base chromosome number, suggested that the inferred ancestral base number should be indicated by the symbol ‘ρ‘ to clearly differ from ‘*x*’. When appropriated, we cited the symbol ‘ρ‘ along with the ‘*x*’, for the sake of clarity.

The ChromEvol software (http://www.tau.ac.il/~itaymay/cp/chromEvol/) uses a likelihood method based on eight types of chromosome number changes along phylogenies. We ran all available models for each phylogenetic proposal (nrDNA and cpDNA under MP and BI) and used the Akaike information criterion (AIC) to select the best model for our dataset. The gain/loss of expected numbers of polyploidy events and the gains and losses of single chromosomes along each branch of the phylogeny were recorded based on the best-fitting model. Chromosome numbers were taken from the literature as well as obtained in the present study. The input data are presented in S2 Table.

## Results

### Molecular data information

The aligned nrDNA dataset consisted of 780 bp with 88 informative characters, and the aligned complete cpDNA dataset consisted of 3014 bp (1813 from the *mat*K + *trn*K locus and 1201 from the *atp*B—*rbc*L intergenic region) with 96 informative characters.

#### Phylogenetic analyses: Maximum Parsimony

**Fig 2:**
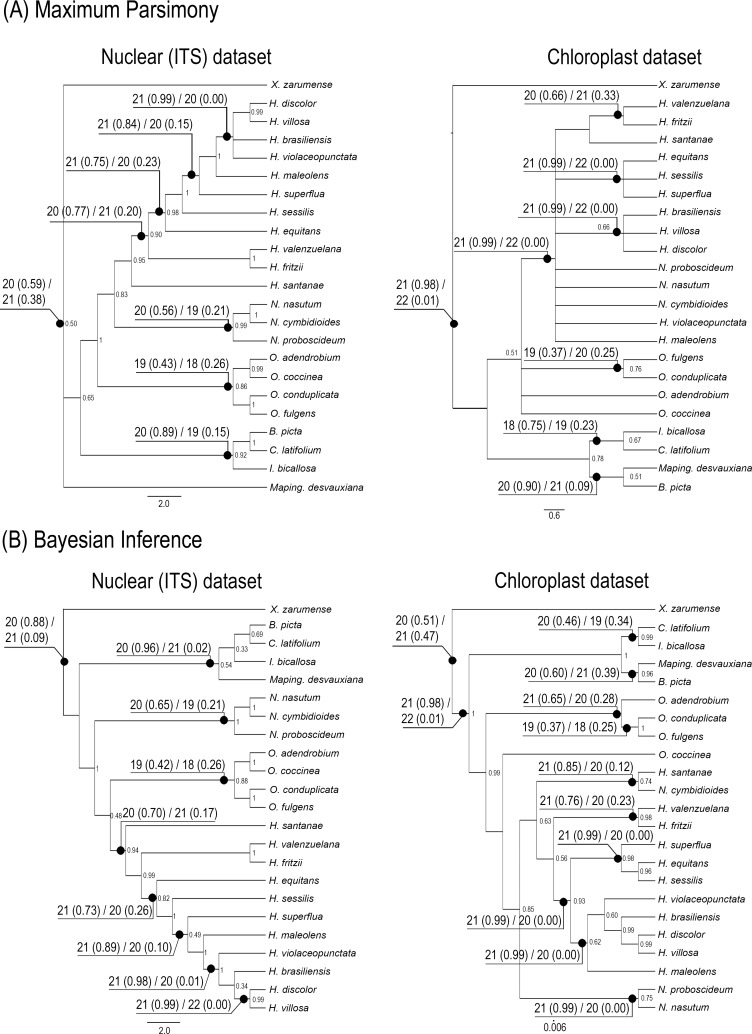
**The strict consensus trees generated using (A) Maximum Parsimony and (B) Bayesian Inference based on nrDNA and cpDNA datasets.** Selected bootstrap values above 0.49 are shown below the branches. For each consensus tree, the results for ancestral base chromosome number evolution estimated by MLE is shown, presenting the two most likely base chromosome numbers (haploid) on selected nodes, followed by the probability in parenthesis.

Based on the most parsimonious trees (MPTs) obtained, some incongruent clades were found between the nrDNA and cpDNA trees, mainly due to the *H*. *equitans*. Additional sequences were obtained for this species to avoid taxonomic errors, but the incongruence was maintained. Only nrDNA recovered the three genera, *Heterotaxis*, *Nitidobulbon* and *Ornithidium*, as monophyletic (Fig 2A). The cpDNA dataset recovered *Nitidobulbon* nested in a comb with *Heterotaxis* and *Ornithidium* as sister of *Nitidobulbon* + *Heterotaxis*. The CI and RI for the individual datasets were CI = 0.571 and RI = 0.734 for nrDNA and CI = 0.518 and RI = 0.718 for cpDNA.

#### Phylogenetic analyses: Bayesian inference

Three models were selected for each molecular marker: TiM3 + G for nrDNA, TPM1uf + G and TIM1 + G for cpDNA (*atp*B and *mat*K + *trn*K, respectively). The tree recovered from the nrDNA dataset contained the three major clades, with strong support for *Nitidobulbon* (PP = 1), placed as sister of *Ornithidium* (moderate support—PP = 0.88) + *Heterotaxis* (marginal strong support—PP = 0.94) (Fig 2B—Nuclear dataset). The separation between *Ornithidium* and *Heterotaxis* received a low support (PP = 0.48). Based on cpDNA, *N*. *cymbidioides* was nested in *Heterotaxis* and *O*. *coccinea* was sister of *Nitidobulbon* + *Heterotaxis* (Fig 2B—Chloroplast dataset). *Marsupiaria*, as previously circumscribed, was nested within *Heterotaxis*, and neither the nrDNA nor cpDNA dataset placed *H*. *valenzuelana* and *H*. *equitans* close to each other. Again, incongruences between nrDNA and chloroplast datasets do not allowed to join both datasets.

#### Chromosome number and genome size

**Fig 3:**
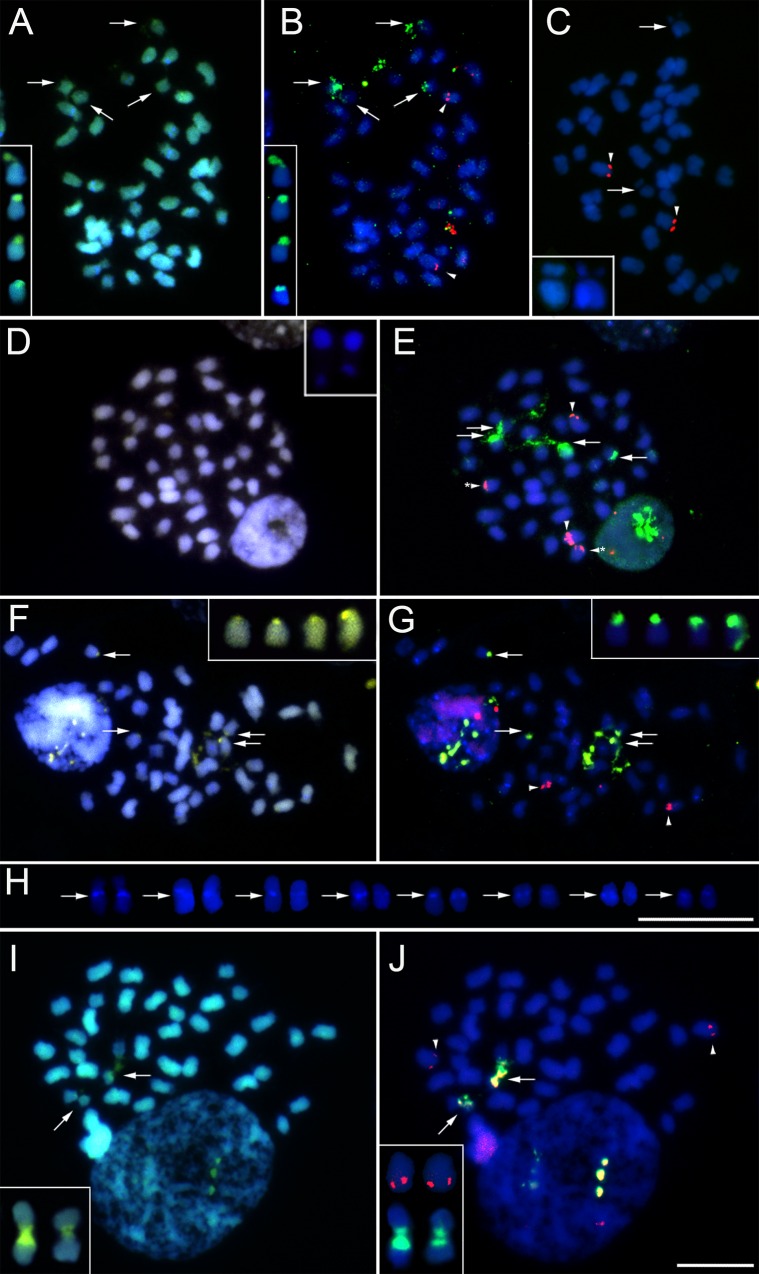
Chromosome analysis of *Heterotaxis*. A-B, *H*. *brasiliensis* (population from Paraty, Brazil); C, *H*. *brasiliensis* (population from Ubatuba, Brazil); D-E, *H*. *equitans*; F-G, *H*. *villosa*; H, *Heterotaxis* chromosomes showing pericentromeric DAPI^+^ bands; I-J, *H*. *valenzuelana*. A, D, F and I: CMA (yellow)/DAPI (blue) banding. H: DAPI^+^ bands. B, E, G and J: *in situ* hybridization using 45S rDNA (green) and 5S rDNA (red). C: 5S rDNA (red). Arrows in A, F and I indicate CMA^0^/DAPI^−^or CMA^+^/DAPI^−^bands. Arrows and arrowheads in B, E, G and J show 45S rDNA and 5S rDNA, respectively. Arrowheads in C show 5S rDNA and arrows indicated the CMA^–^/DAPI^−^chromosome gap. Detail in A, F and I indicate chromosomes with CMA^+^/DAPI^−^bands and in B, G and J, the same chromosomes with 45S rDNA sites (green). Detail in J shows also the chromosome pair with 5S rDNA sites (red). Chromosomes in the inserts in A, B, I and J could be selected from an alternative metaphase. Insets in C and D show the chromosome with CMA^–^/DAPI^−^gap. Bars in H and J represent 10 m.

The 2*n* = 42 was observed in *H*. *brasiliensis*, *H*. *villosa*, *H*. *violaceopunctata*, *H*. *equitans* and *H*. *superflua* and 2*n* = 40 in *H*. *valenzuelana* (Table 3, Fig 3). Regarding the genome size (2C value; see S1 Fig), the six *Heterotaxis* species were divided into two groups (F = 29.7; p < 0.0001): (1) larger genomes, found in *H*. *brasiliensis* (8.64 pg), *H*. *villosa* (8.75 pg) and *H*. *violaceopunctata* (8.51 pg); and (2) smaller genomes, found in *H*. *equitans* (7.70 pg), *H*. *superflua* (7.67 pg) and *H*. *valenzuelana* (7.46 pg) (Table 3). *Mapinguari desvauxiana*, used as an outgroup in the phylogeny, had a 2C = 4.49 pg.

**Table 3:** Karyotype and genome size (2C) data for *Heterotaxis*.

Species	Karyotype^1^					Genome Size	
	2*n*	DAPI^+^	CMA^+^	45S	5S	2C^2^	CV^3^
*Heterotaxis brasiliensis*	42	12–16, pr.	4, ter.	4, ter.	2, ter.	8.64	2.76
					2, int.		
*Heterotaxis violaceopunctata*						8.51	2.43
*Heterotaxis villosa*						8.75	2.57
*Heterotaxis superflua*						7.67	2.65
*Heterotaxis equitans*					4, (2/2—ter/int).	7.70	3.15
*Heterotaxis valenzuelana*	40	-	2, pr.	2, pr.	2, int.	7.46	2.50
Mapinguari desvauxiana*	40	-	2, ter.	2, ter.	2, int. & 2, ter.	4.49	4.26

^1^ pr. = proximal position on the chromosome; int. = intertitial position on the chromosome; ter. = terminal position on the chromosome.

^2^ 2C values are given in picograms.

^3^CV = Coefficient of variation.

*Karyotype data were obtained by [19].

### Karyotype characterization

#### CMA/DAPI banding

The chromosome banding showed four band types: CMA^0^/DAPI^−^(neutral on CMA and dull on DAPI; see arrows in Fig 3A and 3I), CMA^+^/DAPI^−^(bright on CMA and dull on DAPI; see arrows in Fig 3F and inserts in 3A, 3F and 3I), CMA^–^/DAPI^+^ (dull on CMA and bright on DAPI; see arrows in Fig 3H) and CMA^–^/DAPI^−^(dull on both fluorochromes; see arrows and detail in Fig 3C and detail in Fig 3D). Punctual CMA^–^/DAPI^+^ bands were observed in all species in the proximal region of 6–8 chromosome pairs (Fig 3H), which became more evident after *in situ* hybridization (Fig 3B and 3G). However, *H*. *valenzuelana* did not have any DAPI^+^ bands (Fig 3I and 3J).

The four terminal CMA^+^/DAPI^−^bands (see details in Fig 3A and 3F), sometimes were detected as CMA^0^/DAPI^−^bands (Fig 3A) and could be hardly seen in some metaphases (Fig 3D). The bands were observed in the terminal position on two acrocentric chromosome pairs in all species (see details in Fig 3A and 3F), except by *H*. *valenzuelana*, which had two CMA^+^/DAPI^−^bands in the proximal region on a metacentric chromosome pair (Fig 3I). A chromosome pair could be identified by an uncommon CMA^–^/DAPI^−^band in the proximal region (insets in Fig 3C and 3D). The absence of staining with both fluorochromes formed a gap, which was frequently distended, sometimes placing the short arm distant from the long arm.

#### *In situ* hybridization

The 45S rDNA sites were always co-localized with CMA^+^ bands. All species had four terminal 45S rDNA sites on acrocentric chromosomes (Fig 3B, 3E and 3G) with the exception of *H*. *valenzuelana*, which had two proximal 45S rDNA sites on a metacentric chromosome pair (see detail in Fig 3J). We observed two interstitial 5S rDNA sites in most species (Fig 3B, 3G and 3J and detail in 3J); however, *H*. *brasiliensis* from the Ubatuba population (São Paulo State, Brazil) had the two 5S rDNA sites in a terminal position (Fig 3C). Moreover, *H*. *equitans* had four sites: two interstitial sites in one chromosome pair and two terminal sites in another (Fig 3E).

### Reconstruction of ancestral chromosome number

**Fig 4:**
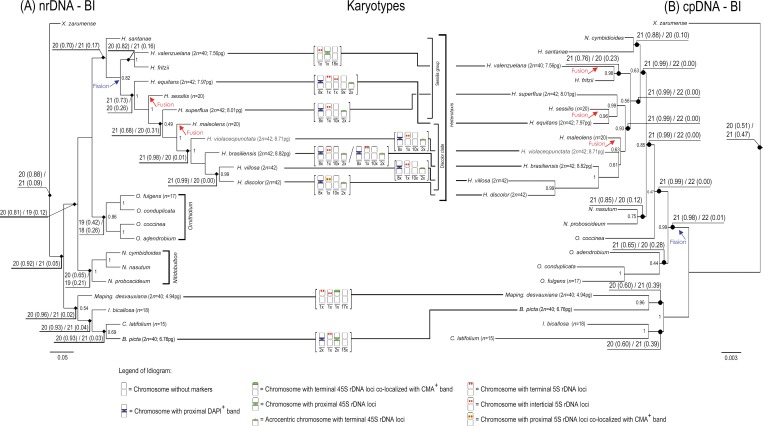
**Majority rule consensus tree generated using Bayesian Inference based on the (A) nrDNA and (B) cpDNA datasets, presenting also the ancestral base chromosome number evolution estimated by MLE and karyotypes obtained.** Blue arrow indicate a probably point of chromosome gain (supposed fission), while red arrow indicate a probably point of chromosome loss (supposed fusion). The two most likely base chromosome numbers (haploid) are indicated on selected nodes, followed by the probability in parenthesis. Genome sizes are indicated at the terminal, after the species name. An idiogram for species with karyotype data is shown after the terminal. Data for *B*. *picta* were determined by [25]. Selected PP values above 0.49 are shown on the nodes.

Due to the incongruences between nrDNA and cpDNA datasets we used the four phylogenetic proposals independently for ancestral reconstruction of the basic chromosome number (Fig 2). The results for nrDNA and cpDNA under BI are compared in Fig 4 with idiograms for all species analysed. For the four phylogenetical proposals, the best-fitting ML model was the combination of constant gain and loss without duplication (*i*.*e*., just dysploidy events) for the four phylogenetic hypotheses used (Table 4).

**Table 4:** Likelihood estimates and AIC scores for the four phylogenetical proposals tested using the ChromEvol software.

	Maximum Parcimony		Bayesian Inference	
MODEL	Log-likelihood	AIC	Log-likelihood	AIC
nrDNA				
CONST_RATE	-14.92	35.84	-15.73	37.46
CONST_RATE_DEMI	-14.92	35.84	-15.73	37.46
CONST_RATE_DEMI_EST	-14.92	37.84	-15.73	39.46
CONST_RATE_NO_DUPL	-14.92	33.84	-15.73	35.46
LINEAR_RATE	-15.08	40.15	-15.89	41.77
LINEAR_RATE_DEMI	-15.08	40.15	-15.89	41.77
LINEAR_RATE_DEMI_EST	-15.08	42.15	-15.89	43.77
LINEAR_RATE_NO_DUPL	-15.08	38.15	-15.89	39.66
cpDNA				
CONST_RATE	-22.41	50.81	-19.46	44.92
CONST_RATE_DEMI	-22.41	50.81	-19.46	44.92
CONST_RATE_DEMI_EST	-22.41	52.81	-19.46	46.92
CONST_RATE_NO_DUPL	-22.41	48.81	-19.46	42.92
LINEAR_RATE	-22.77	55.55	-19.75	49.50
LINEAR_RATE_DEMI	-22.77	55.55	-19.75	49.50
LINEAR_RATE_DEMI_EST	-22.77	57.61	-19.75	51.6
LINEAR_RATE_NO_DUPL	-22.77	53.55	-19.75	47.5

When using phylogenetic hypotheses based on nrDNA, the best-fitting model suggested *x* = 20 (or ρ = 20, if following nomenclature suggested in [45]) as the inferred ancestral basic chromosome number of *Heterotaxis*. However, *x* = 21 (or ρ = 21) was suggested when using phylogenetic hypotheses based on cpDNA (Figs 2 and 4). In three out four tested phylogenetic hypothesis, *x* = 20 (or ρ = 20) was the inferred as the ancestral state for the whole group of species used in the phylogeny. Besides that just nrDNA datasets recovered the three genera as monophyletics, the difference among nrDNA x cpDNA phylogenetic hypothesis is when the first fission event occurred: (1) if in the beginning of *Heterotaxis* diversification, with two subsequent fusions in *H*. *maleolens* and *H*. *sessilis*, as suggested by nrDNA datatsets; or (2) before *Heterotaxis* diversification and the three fusions events occurred inside *Heterotaxis* genus, reducing the chromosome number from 2*n* = 42 to 2*n* = 40 in *H*. *maleolens*, *H*. *sessilis* and *H*. *valenzuelana*, as suggested by cpDNA datasets.

## Discussion

The interpretation of the pattern of chromosome evolution detected here is supported by a set of phylogeny hypothesis suggesting the occurrence of chromosome fission and subsequent chromosome fusion. The chromosome data obtained from species of *Heterotaxis* are consistent with the variation observed in other Orchidaceae genera: frequent dysploidy (caused by chromosome fission and fusion), 5S rDNA position/number changes and a diversity of heterochromatic bands revealed by CMA/DAPI banding.

### Phylogenetic relationships

#### Genus *Heterotaxis*

Traditionally, the *Heterotaxis* species were organized into two major morphological groups–Sessilis and Discolor [18, 29, 30]. Both groups were recovered here, Sessilis as a grade and Discolor as a clade.

1. Sessilis grade: This group of species comprised *H*. *santanae*, *H*. *valenzuelana*, *H*. *fritzii*, *H*. *equitans*, *H*. *sessilis* and *H*. *superflua* (Fig 4), all of which are conspicuously succulent with small vegetative, floral organs and small GSs, as well.
2. Discolor clade: This clade is well supported (see both nrDNA phylogeny trees in Fig 2) and morphologically characterized by robust vegetative and floral parts and the presence of a three lobed lip with an ovate apex. This clade contains five robust species–*H*. *maleolens*, *H*. *violaceopunctata*, *H*. *brasiliensis*, *H*. *villosa* and *H*. *discolor* which present also a large GS (Fig 4).

#### Genera *Ornithidium* and *Nitidobulbon*

The organization of cpDNA trees probably reflect previous hybridization event between *Heterotaxis* and *Ornithidium* and *Nitidobulbon* species. The proximity of *Nitidobulbon* and *Heterotaxis* is reflected by morphological similarities between genera and the three species currently grouped in *Nitidobulbon* were traditionally included in *Heterotaxis* and, even nowadays, are sometimes misidentified [23, 48, 49].

### Karyotypes in *Heterotaxis*

In this study, we report new chromosome numbers for *H*. *brasiliensis*, *H*. *superflua* and *H*. *equitans* and also confirm previous reports for *H*. *violaceopunctata* and *H*. *valenzuelana*. However, we found discrepancies between the chromosome number previously reported for *H*. *villosa* (*n* = 20) (Table 1, [21]) and that obtained from our analysis (2*n* = 42; Table 3). Such intraspecific karyotype variation suggests either the occurrence of counting errors/misidentifications or occurrence of different cytotypes; *i*.*e*., populations with divergent karyotypes. Such difference could be due to polyploidy, aneuploidy or dysploidy rearrangements [1–3], what could be the case of *H*. *villosa*.

The presence of multiple cytotypes, specially dysploidy cytotypes, seems to be neglected in taxonomic and ecological studies [4, 5, 8, 46, 50]. However, reports of such variation among populations are common [51–52], even in taxonomic groups in which dysploidy is considered rare, such as in subfamily Mimosoideae (Leguminosae; 1.46% of species show dysploidy) [53–54]. Unfortunately, Blumenschein & Paker [21] did not deposit any vouchers of the analysed material; therefore, the possibility of misidentification should not be ruled out, especially considering the challenging taxonomy of this genus [18, 23, 30, 55].

### Karyotype and GS evolution in *Heterotaxis*

Recently, Escudero et al. [11] analysed chromosome gains and losses in 15 angiosperms clades, including the subtribe Orchidinae (Orchideae: Orchidaceae). The authors proposed dysploidy as a predominant mechanism in Orchidinae, as previously suggested for other subfamilies of Orchidaceae [56, 57]. Dysploidy is traditionally suggested as the cause of chromosome number variation in subfamily Cypripedioideae [58], in the genus *Paphiopedilum* Pfitzer [59] and in tribe Neottieae, including *Cephalanthera* Rich. [60, 61], *Epipactis* Zinn and *Neottia* Guett. [62]. These studies suggest that dysploidy plays a key role in the chromosome evolution of Orchidaceae [56].

The variation in chromosome number detected in *Heterotaxis* also appears to be caused by dysploidy. However, the separation between taxonomic groups, grade Sessilis and clade Discolor, is more likely caused by repetitive DNA variation, increasing the GS in the clade Discolor. It is traditionally assumed that plants with large GS present large morphological traits [63]. However, this hypothesis could be confirmed just in small groups of related species and, when using higher phenotypic scales, this relationship is often reduced [64, 65].

Our findings support the inference that the dysploidy variation was primarily caused by chromosome fission in an ancestral presenting 2*n* = 40 and a proximal 45S rDNA site on a metacentric chromosome pair. Such species, after a fission in the 45S rDNA site, originated species with 2*n* = 42 and two acrocentric chromosome pairs with terminal 45S rDNA sites.

Despite some doubt about when the chromosome fission occurred, it is certainly that a fission event happened just before *Heterotaxis* diversification (cpDNA phylogeny) or at the beginning of *Heterotaxis* diversification, after separation of *H*. *santanae*, *H*. *fritzii* and *H*. *valenzuelana* (nrDNA phylogeny). However, considering that nrDNA dataset provides a more robust phylogeny, we can assume *x* = 20 (or ρ = 20) to *Heterotaxis*. The occurence of one fission originated the 2*n* = 42 and the two subsequent fusion, in *H*. *sessilis* and *H*. *maleolens*, restored the 2*n* = 40 in these two species. The hypothesis of chromosome evolution presented here diverge of both White’s hypothesis [12] and Minimal Interaction Theory [13–15], but proposed a more dynamic karyotype evolution with both event occurring repeated times.

Actually, some chromosome characteristics facilitated the occurrence of repeatedly fusion-fission chromosome events. For example, the chromosome bouquet configuration during meiosis, *i*.*e*. telomere clustered together at one side and centromeres clustered at the opposite side during chromosome pairing in meiosis [66], facilitates chromosome rearrangements [67]. Such configuration facilitate chromosome centromeres fission, as well as, fusion of chromosome terminals. Moreover, the 45S rDNA is a fragile site in the chromosome, susceptible to breaks and unions, and after a chromosome rearrangement, the unbound terminals can join, facilitating chromosome fusion [66]. These small breaks and chromosome fusion events are common and can occur many times in the same chromosome site. Therefore, such rearrangements could be responsible for a large proportion of the chromosome number variation observed in the Orchidaceae.

However, rearrangements other than fusion/fission events are also responsible for modelling the karyotype. Here, inversions seem to play an important role in chromosome evolution. It is generally accepted that 5S rDNA sites vary less in number and position than do 45S rDNA sites [68]. However, the Orchidaceae seems to be an exception, with their 5S rDNA sites being highly variable in number, position and sequence [25, 69, 70]. The duplication of the 5S rDNA site in *H*. *equitans* and the site position changes detected in one population of *H*. *brasiliensis* support inversion as the second more important chromosome rearrangement in *Heterotaxis* karyotype evolution.

The variation in 5S rDNA position observed in *H*. *brasiliensis* is likely the consequence of a paracentric inversion, moving the sites from an interstitial position to a terminal position. In *H*. *equitans*, one of the points of chromosome breakage (allowing the chromosome inversion to occur) was probably inside the 5S rDNA site and, after inversion, the rearrangement originated a second site. In this sense, inversion happened twice during *Heterotaxis* evolution and seems to be frequent in the Orchidaceae, as observed in *Cephalanthera* [61] and *Christensonella subulata* (Lindl.) Szlach., Mytnik, Górniak & Śmiszek [25]. In addition to dysploidy, inversion is probably a recurrent chromosome rearrangement modelling Orchidaceae karyotypes.

## Conclusion

The refined karyotype characterization analysed under a phylogenetic context reinforces the dysploidy importance in the chromosome evolution and the GS importance in the separation of groups of species. If in one hand, larger GS coincides with larger morphological structures; in the other hand the chromosome number variation seems to be a very dynamic rearrangement not related with groups separation in *Heterotaxis*. Following the well resolved phylogenetic hypothesis, nrDNA under MP and BI, it is likely that 2*n* = 40 is an ancestral state, while 2*n* = 42, observed in the majority of the species, is likely to be a derived condition. However, chromosome fusions restored the ancestral condition in *H*. *maleolens* and *H*. *sessilis*. The cpDNA suggested three fusion event in *Heterotaxis* with 2*n* = 42 as ancestral, but cpDNA has a lower phylogenetic resolution when compared with nrDNA hypothesis.

In addition to dysploidy, inversions appear to take part in modelling Orchidaceae karyotypes, moving 5S rDNA sites and sometimes duplicating them. The identification of chromosome rearrangements presented here reinforces the importance of a phylogenetical framework and statistical methods for ancestral state reconstruction, shedding light on chromosome rearrangements throughout species diversification.

## Supporting Information

S1 Fig Flow cytometry histograms. Representative flow histograms of relative fluorescence (X–axis) obtained after isolation of nuclei from *Heterotaxis* and *M*. *desvauxiana* with internal calibration standards, *Zea mays* and *Vicia fava*, respectively. Peaks are identified in each Fig A, *Zea mays;* B, *H*. *brasiliensis;* C, *H*. *villosa*; D, *H*. *violaceopunctata*; E, *H*. *equitans*; F, *H*. *superflua*; G, *H*. *valenzuelana*; H, *Vicia fava*; I, *Mapinguari desvauxiana*.
(TIF)

S1 Table Genbank accession for nuclear and chloroplast DNA sequences used in the phylogeny. All sequences were published by [29].
(DOC)

S2 Table Haploid chromosome number (*n*) mapped along the phylogeny. Missing data are represented as "x".
(DOC)
