# Supplementary figures and images for: Detecting Mechanisms of Karyotype Evolution in Heterotaxis (Orchidaceae)
Source: PLoS One. 2016 Nov 10;11(11):e0165960. doi: 10.1371/journal.pone.0165960 (PMC5104408; doi:10.1371/journal.pone.0165960)

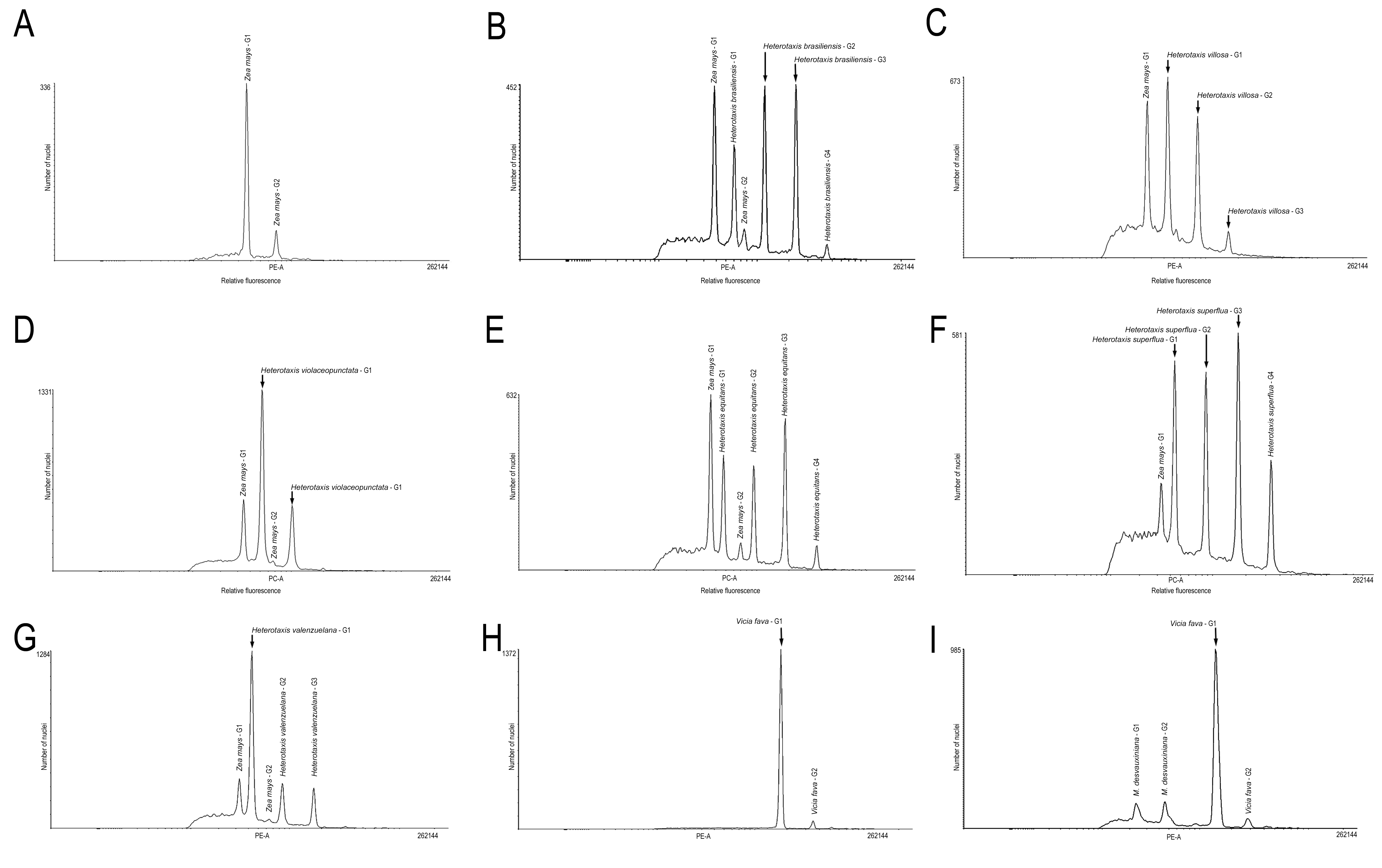

Supplement: S1 Fig — Representative flow histograms of relative fluorescence (X–axis) obtained after isolation of nuclei from Heterotaxis and M. desvauxiana with internal calibration standards, Zea mays and Vicia fava, respectively. Peaks are identified in each Fig A, Zea mays; B, H. brasiliensis; C, H. villosa; D, H. violaceopunctata; E, H. equitans; F, H. superflua; G, H. valenzuelana; H, Vicia fava; I, Mapinguari desvauxiana. (TIF) [file pone.0165960.s001.tif]
