# Supplementary material for: Detecting Mechanisms of Karyotype Evolution in Heterotaxis (Orchidaceae)
Source: PLoS One. 2016 Nov 10;11(11):e0165960. doi: 10.1371/journal.pone.0165960 (PMC5104408; doi:10.1371/journal.pone.0165960)
Supplement: S1 Table — All sequences were published by [29]. (DOC) [file pone.0165960.s002.doc]

Table S1. Genbank accession for nuclear and chloroplast DNA sequences used in the phylogeny. All sequences were published by [29].

| Clade | Species | Molecular markers | | |
| --- | --- | --- | --- | --- |
| *Heterotaxis* |  | ITS | *mat*K + *trn*K | *atp*B-*rbc*L spacer |
|  | *Heterotaxis discolor* | DQ210181 | DQ210711 | DQ209488 |
|  | *Heterotaxis villosa* | DQ210202 | DQ210732 | DQ209509 |
|  | *Heterotaxis brasiliensis* | DQ210155 | DQ210687 | DQ209465 |
|  | *Heterotaxis maleolens* | DQ209978 | DQ209856 | DQ209295 |
|  | *Heterotaxis violaceopunctata* | DQ210146 | DQ210678 | DQ209457 |
|  | *Heterotaxis superflua* | DQ210157 | DQ210689 | DQ209466 |
|  | *Heterotaxis sessilis* | DQ209986 | DQ209862 | DQ209303 |
|  | *Heterotaxis valenzuelana* | DQ210170 | DQ210700 | DQ209477 |
|  | *Heterotaxis fritzii* | DQ210497 | DQ209969 | DQ209781 |
|  | *Heterotaxis equitans* Koehler 0141 | DQ210151 | DQ210683 | DQ209461 |
|  | *Heterotaxis equitans* IBt 979 | KX822792 | KX822790 | KX822788 |
|  | *Heterotaxis equitans* IBt P3931 | KX822793 | KX822791 | KX822789 |
|  | *Heterotaxis santanae* | DQ210526 | DQ209973 | DQ209808 |
| *Nitidobulbon* | *Nitidobulbon nasuta* | DQ210169 | DQ210699 | DQ209476 |
|  | *Nitidobulbon cymbidiodes* | DQ209987 | DQ209863 | DQ209304 |
|  | *Nitidobulbon proboscidea* | DQ209979 | DQ209857 | DQ209296 |
| *Ornithidium* | *Ornithidium adendrobium* | DQ210214 | DQ210741 | DQ209519 |
|  | *Ornithidium conduplicata* | DQ210041 | DQ209889 | DQ209356 |
|  | *Ornithidium coccinea* | DQ210009 | DQ209875 | DQ209324 |
|  | *Ornithidium fulgens* | DQ210472 | DQ209968 | DQ209755 |
| Out group | *Brasiliorchis picta* | DQ210190 | DQ210720 | DQ209497 |
|  | *Mapinguari desvauxiana* | DQ210206 | DQ210736 | DQ209513 |
|  | *Inti bicallosa* | DQ210517 | DQ210998 | DQ209800 |
|  | *Cryptocentrum latifolium* | DQ210332 | DQ210831 | DQ209619 |
|  | *Xylobium zarumense* | AF239339 | AF239435 | DQ209578 |
