# Supplementary material for: Detecting Mechanisms of Karyotype Evolution in Heterotaxis (Orchidaceae)
Source: PLoS One. 2016 Nov 10;11(11):e0165960. doi: 10.1371/journal.pone.0165960 (PMC5104408; doi:10.1371/journal.pone.0165960)
Supplement: S2 Table — Missing data are represented as "x". (DOC) [file pone.0165960.s003.doc]

Table S2. Haploid chromosome number (*n*) mapped along the phylogeny. Missing data are represented as "x".

>'Heterotaxis_santanae'

x

>'Heterotaxis_equitans'

21

>'Heterotaxis_superflua'

21

>'Heterotaxis_discolor'

21

>'Heterotaxis_villosa'

21

>'Heterotaxis_violaceopunctata'

21

>'Heterotaxis_brasiliensis'

21

>'Heterotaxis_maleolens'

20

>'Heterotaxis_sessilis'

20

>'Heterotaxis_valenzuelana'

20

>'Heterotaxis_fritzii'

x

>'Ornithidium_fulgens'

17

>'Ornithidium_conduplicata'

x

>'Ornithidium_adendrobium'

x

>'Ornithidium_coccinea'

x

>'Nitidobulbon_cymbioides'

x

>'Nitidobulbon_nasuta'

x

>'Nitidobulbon_proboscidea'

x

>'Mapinguari_desvauxiana'

20

>'Inti_bicallosa'

18

>'Cryptocentrum_ latifolium'

15

>'Brasiliorchis_ picta'

20

>'Xylobium_zarumense'

x
